# Distortions of space and time in and around objects and events

**DOI:** 10.3758/s13423-025-02848-6

**Published:** 2026-02-26

**Authors:** Sami R. Yousif, Brynn E. Sherman

**Affiliations:** Department of Psychology, Ohio State University, Columbus, OH USA

**Keywords:** Space, Time, Objects, Events

## Abstract

Our perceptual experience is not a veridical representation of the world around us. We perceive structure in the form of *objects* and *events*, and this structure has consequences. Like gravity distorts space and time in the physical world, objects and events distort space and time in our minds, resulting in a plethora of illusions that observers can readily appreciate for themselves. Moreover, many of these distortions of space (caused by objects) and time (caused by events) appear to be perfect analogs of one another—suggesting a deep relationship between the representation of space and time, objects and events. Here we review dozens of illusions of space and time, discussing how they relate to one another and what these relations reveal about the organization of perceptual and mnemonic systems in the human mind.

Look out at the world around you. What do you see? Perhaps the denizens of a coffee shop, a crowd of people waiting in line, or a car parked just outside. *Listen* to the world around you. What do you hear? A steady drone of background noise, an occasional laugh, the hums and whirs of traffic through the glass. You see and hear not arbitrary “stuff” but *structure* in the world around you. That is, you see objects, and you experience events. In a “blooming, buzzing” world, objects and events are the structure. Like atoms or molecules are the “units” of matter, objects and events are the units of experience.

The demarcation of our experience into structured units is not without consequence: Like gravity bends space and time in the physical world, objects and events bend space and time in the mind—resulting in a wide range of remarkable illusions. For example, simply placing two dots inside of a rectangle causes them to be perceived as farther from each other (compared with two comparable dots without a bounding rectangle). This, aptly, is known as “object-based warping” (Vickery & Chun, 2010; see Fig. 1A). Events, in time, rather than objects in space, exhibit the very same warping (Goh et al., 2025).

**Fig. 1:**
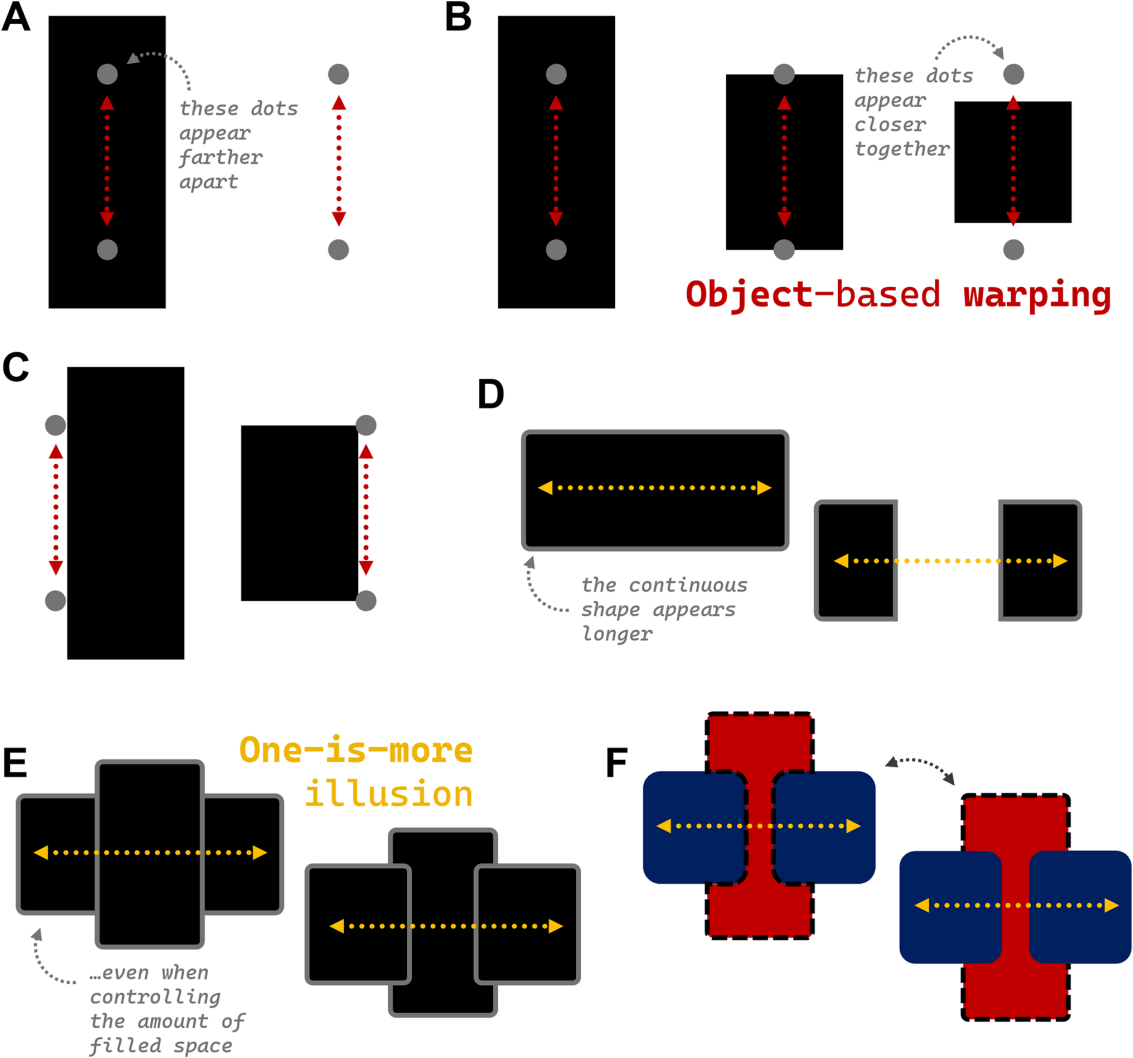
Various illustrations of object-based warping and the one-is-more illusion. **A** The canonical object-based warping effect, wherein dots within the rectangle are perceived as further apart. **B** Variations of object-based warping. **C** Object-based warping near rather than within objects. **D** The canonical one-is-more illusion, wherein continuous entities are perceived as ~30% longer than discrete entities. **E** The one-is-more illusion persisting through occlusion. **F** An example of the bi-stable “apple core” image used to show the one-is-more illusion is evident even when visual input is perfectly equated (dashed lines are to highlight possible interpretations; they are for illustrative purposes only). In all cases, the relevant extents are highlighted with dotted lines; note that these illusions appear stronger without these guiding lines. Versions of these demos without guiding lines can be found at https://www.spatialcognitionlab.org/spacetime (Color figure online)

But how exactly are space and time being distorted in these cases? Is it merely the space and time within objects and events that are distorted, or are the areas *around* them also distorted? Are these effects really about objects and events per se? Finally, what might such distortions reveal about the relation between the representation of space and time, objects and events? The aim of this paper is to review work on spatial and temporal distortions that arise from object and event structure and answer these questions along the way.

## Defining objects and events

Objects and events, like so many other things, may only be defined on a know-it-when-you-see-it basis (see Scholl, 2001; Yates et al., 2023). Roughly, though, objects are structured *spatial* entities. A lamp is an object because it is a coherent physical entity that moves together and has a single purpose. Objects can be composed of other objects: A lamp consists of objects like a lightbulb and a lampshade, which are also coherent physical entities that move together and have a single purpose. They can be small (like a coffee mug) or large (like a stadium). They not only can compose into other objects, they can also be *contained* by other objects; their organization can be hierarchical.

Whereas objects refer to *spatial* structure, events refer to *temporal* structure. In the same way that an object is a coherent physical entity, an event is a coherent temporal entity, bounded by a beginning and ending. Events, like objects, are hierarchical in that events can be nested within larger events (e.g., the event of “brushing teeth” can exist within the larger event of “getting ready”). Because of these deep structural similarities, events are often considered to be the temporal analog of objects (e.g., De Freitas et al., 2014; Yates et al., 2023).

There is much to say about how to define objects and events more precisely (see, e.g., Scholl, 2001; Yates et al., 2023). We will sidestep those (deep, interesting) questions for now. Instead, we will focus on the *consequences* of objecthood and eventhood and how it is that they distort the space and time around them.

## Distortions of space in and around objects

One of the core functions of the human mind and brain is to represent the *space* around us. To avoid an obstacle, to grab a coffee mug, to align two graphs on a slide, we need to represent the locations of and relations between objects in space. Yet space itself can be surprisingly and powerfully distorted by the presence of objects. In this section, we review the ways in which objects can lead to robust and systematic distortions of perceived space and what such distortions reveal about the ways the mind parses and represents our spatial world.

### Distortions of one-dimensional space around the bounds of an object

We will begin with *object-based warping*. Simply, two dots within an object appear farther apart than two comparable dots that occupy empty space (Vickery & Chun, 2010; see Fig. 1A; stronger versions of these demos and all other demos can be found at https://www.spatialcognitionlab.org/spacetime). This phenomenon has been (speculatively) linked to *object-based attention*, whereby attention spreads not like a spotlight over space, but over structured objects (Scholl, 2001). But how exactly does the spread of attention warp the sense of space? Though Vickery and Chun (2010) shy away from offering a definitive explanation of this phenomenon, they speculate that either “object-based warping may be related to attention, which involuntarily spreads across or arbitrarily selects entire surfaces of incidentally selected regions belonging to objects,” or “the visual system may devote an exaggerated cortical representation to visual regions perceived as belonging to a figure’s surface, because of the primacy of objects in perceptual computations” (p. 1763). These explanations are complicated, however, by variations of the illusion, wherein, for instance, dots placed on opposite edges of an object or just outside either bound of the object appear *closer* than dots occupying empty space (see Fig. 1B; for these and many other variations, see Lebed et al., 2023).

Lebed and colleagues (2023) discuss at length how object-based warping might simultaneously explain the canonical *expansion* of space when probes are placed within an object as well as the *compression* of space when probes are placed around an object: Perhaps these opposing effects reflect the center-surround nature of attentional selection, in that regions around the focal region are suppressed (thus resulting in compression). Or perhaps object boundaries serve as landmarks or “attractor points” such that dots inside an object are pulled toward the boundaries and end up seeming farther from one another whereas dots outside, also pulled toward the boundaries, end up seeming closer. Or perhaps these are two distinct phenomena: The canonical expansion effects may be caused by distinct mechanisms from the compression effects. For now, these remain open questions.

One way to begin answering these questions is to understand what space is actually being warped in the first place. Vickery and Chun (2010) assess the warping of space via probe dots placed either superimposed on salient objects or in empty space. Lebed and colleagues (2023) go one step further by placing the probe dots outside of the boundary of the object but not along the primary axis of the shape (e.g., horizontally offset to the right or left of object; see Fig. 1C). Interestingly, they find that object-based warping applies to space just outside of objects. In fact, both expansion and compression effects get gradually weaker as the probe dots move farther from the central axis, as if the space is continuously warped in the vicinity of salient objects (rather than strictly within the bounds of an object). On a strictly object-based account, such continuous distortions might be viewed as mysterious since they extend beyond the boundary of the object itself. Similarly, these continuous effects of spatial distance imply that the boundary itself is not critical for the illusion; if it was, one might expect the illusion to break for any object outside the bounds of the shape.

If the space around objects is indeed continuously warped, then perhaps we should expect that the length of objects themselves (rather than the extent between two probe dots) is similarly distorted. Indeed, this seems to be the case, as you can readily see for yourself in Fig. 1D. Here, a single, continuous rectangle is perceived as longer from edge-to-edge than two, discrete rectangles—a phenomenon referred to as the *one-is-more illusion* (Yousif & Scholl, 2019), so named because *one thing* is consistently perceived as greater in extent than any collection of things. The resulting distortions are not small, as viewers can surely appreciate for themselves: People tend to perceive the continuous entity as about 30% longer overall. Looking at this figure, an obvious possibility is that observers perceive the difference in white space between the two displays. Yet the one-is-more-illusion persists even in cases of occlusion such that the overall amount of white space in the display is equated. In Fig. 1E, you can see in one case a single, horizontal rectangle occluded by a vertical rectangle, and in another case two separate discrete rectangles in front of a vertical rectangle. Both figures contain the same amount of white space, and yet the seemingly continuous rectangle behind the occluder nevertheless appears greater in length. In fact, the one-is-more illusion applies to stimuli that are *perfectly* controlled in the sense that participants evaluated the exact same stimuli with respect to one of two bi-stable percepts (see Fig. 1F). Here, observers were first asked to appreciate two different interpretations of the presented objects: Two smaller blue rectangles in front of a vertical red rectangle *or* one single blue rectangle behind a red “apple core” shape. Once observers were able to acknowledge both interpretations, they were asked to indicate which of the two interpretations seemed longer in horizontal extent. Even in this unnatural task, 70% of participants reported that the single blue rectangle was longer.

Even this effect might be explained by a figure-ground effect, however: The presence of an occluder may cause the putatively continuous object to be perceived as farther away (i.e., because it is placed behind another object). If the object is perceived as farther away, it may be seen as larger because the visual system tries to compute its *true* size, rather than its retinal size. Yousif and Scholl (2019) account for this possibility in two ways. First, they asked observers to evaluate the edge-to-edge extent of two separate rectangles behind an occluder. But, critically, those two rectangles either moved in sync or out of sync in the vertical dimension—thus, in the former cases, appearing as one continuous entity, and in the latter cases, appearing as two discrete entities. Both cases involved the same degree of occlusion (and presumably, then, the same inferences about depth), yet the single continuous rectangle was again perceived as longer. Second, a version of the study was conducted in which depth information was precisely manipulated using a stereoscopic headset. Even when the continuous, occluded object was in fact immediately behind the occluder (per the discrepancy in position presented to each eye), the illusion persisted.

Yousif and Scholl (2019) argue that these effects reflect the same kind of spatial distortion discovered by Vickery and Chun (2010), except that their demonstrations show that it is the space of the objects themselves that is being distorted. Note that this is especially surprising when you consider the fact that dots placed on or near object boundaries exhibit compression, rather than expansion (Lebed et al., 2023). If the continuous space of an object is expanded, then why would probes placed precisely at each boundary be compressed? Right now, there is no clear answer to this question (for a possible explanation, see Experiment 4 of Lebed, 2022). Yet it is mysteries like these that suggest that, for as much that is known about these distortions, there is much left to understand.

**Fig. 2:**
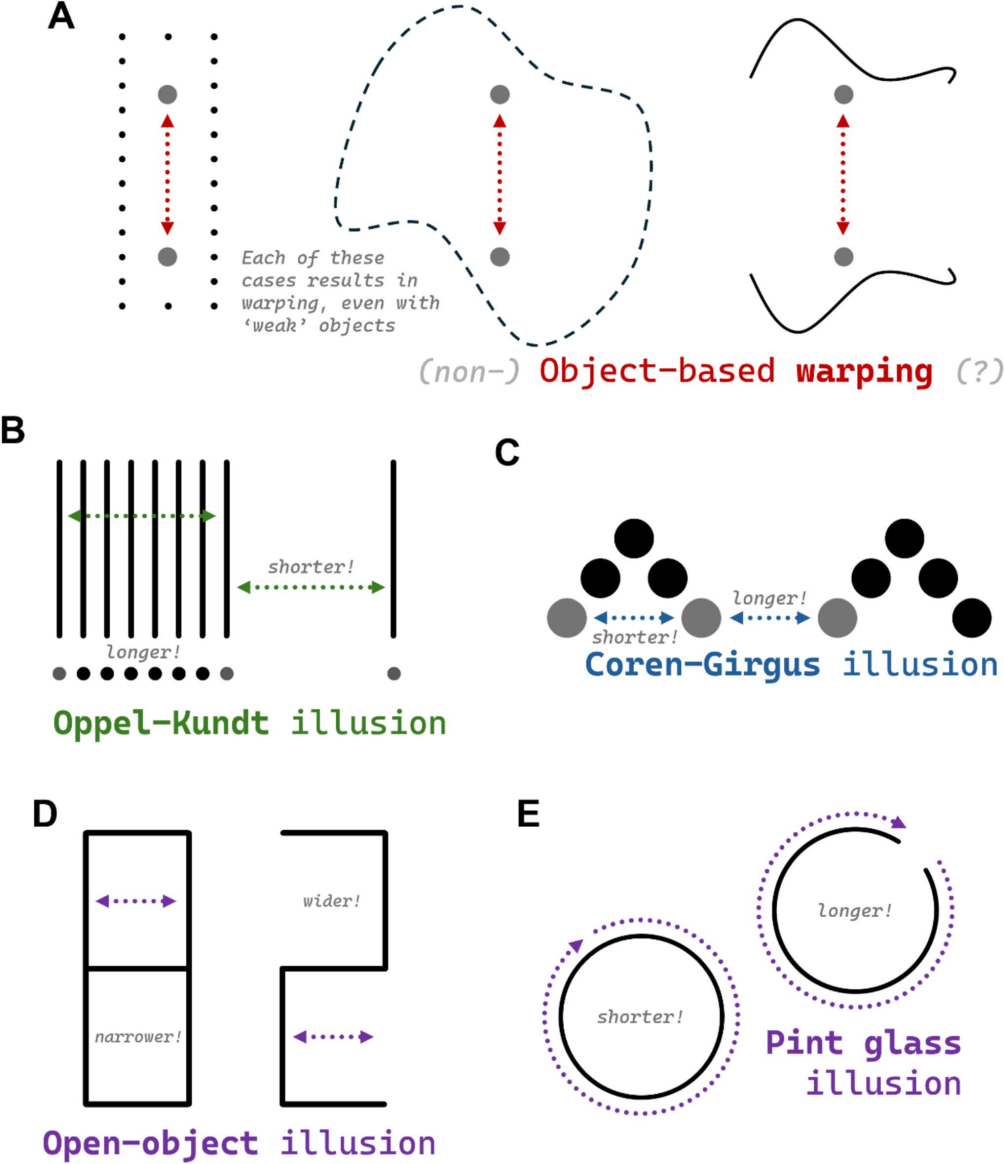
Other illusions of spatial extent. **A** Variations of object-based warping, without clear objects. **B** Two examples of the Oppel–Kundt illusion, wherein the extent across many lines/dots is perceived as greater than the extent across empty space. **C** The Coren–Girgus illusion, wherein dots between sets are perceived as farther apart. **D** The open-object illusion. **E** The pint glass illusion. In all cases, the relevant extents are highlighted with dotted lines; note that these illusions appear stronger without these guiding lines. Versions of these demos without guiding lines can be found at https://www.spatialcognitionlab.org/spacetime (Color figure online)

All of the above work assumes that the observed distortions of space are somehow related to the spread of attention. How could we know for sure? One approach might be to test stimuli that are like the relevant tested stimuli, but without complete, bounded objects. Baker and colleagues (2024) do exactly that. Across six unique experiments, they show a similar expansion of space even when probe dots are placed in between two independent contours, for instance (see Fig. 2A). Their demonstrations are thorough and compelling. Clearly, object-based warping does not depend on the presence of strictly defined objects. However, it is not clear that for something to be “object-based” it must depend solely on such rigidly defined, unambiguous objects. In fact, object-based effects are often correlated with spatial structure such that clearer cues to objecthood result in stronger effects; they are rarely all-or-none (see Feldman, 2007; Marino & Scholl, 2005; Yousif & Scholl, 2019). Baker and colleagues may be correct to note that object-based warping is flexible, but we do not see these findings as in any way undermining the reality of the phenomenon. *Something* about the structure of space distorts our perception of it, whether that is objects or something object-like.

Three other visual illusions are worth mentioning. The first is the *Oppel–Kundt illusion* (Kundt, 1863; for a more recent exploration, see Mikellidou & Thompson, 2014; see two variations in Fig. 2B). In this illusion, additional vertical bars placed in between two bounding vertical bars cause the extent between two bounding bars to be perceived as greater. Note, however, that the Oppel–Kundt illusion is not just a “more-is-more” bias, because, as we have already discussed, *one* object is perceived as the “most” (Yousif & Scholl, 2019). Thus, it is almost as if adding additional bars creates additional structure, causing the collection of objects to, counterintuitively, appear more like one single entity. Viewed this way, the expansion of space resulting from added bars has an object-based explanation and thus may be directly related to object-based warping and the one-is-more illusion.

The second is the *Coren–Girgus illusion*, wherein visually grouped dots are perceived as *closer* together than comparable ungrouped dots (Coren & Girgus, 1980; see Fig. 2C). At a glance, this illusion seems at odds with both object-based warping and the one-is-more illusion in that the more structured entities are perceived as farther apart than the less structured entities. However, some details are worth noting here. First, of course, the groups of entities in the Coren–Girgus illusion are still made up of discrete objects; it is not clear what should be made of these “hierarchical” visual objects that are composed of otherwise discrete entities. Second, it is worth noting that the Coren–Girgus illusion is *much* more subtle than object-based warping and certainly much more subtle than the one-is-more illusion (which results in spatial distortions as large as 30%). Thus, while it seems inevitably true that these illusions will involve some similar grouping/attentional mechanisms, they are in very different ballparks with respect to their phenomenological consequences.

The third is the *open-object illusion*, whereby “open objects” (i.e., ones that do not form a fully bounded polygon) appear to be noticeably larger (Makovski, 2017; see Fig. 2D). Makovski (2017) speculates that this illusion may reflect a kind of “boundary extension” (see Intraub & Richardson, 1989) but offers no definitive explanation of the illusion. One seemingly unexplored possibility, though, is whether this truly reflects the openness of the object or the lower-level features that compose the open versus closed objects in the first place. In Fig. 2D, for instance, the closed object has more vertical lines, which may draw more attention along that vertical axis and thus reduces perceived length along the opposite axis. This is a possibility that could be explored further. However, it is worth noting that there are other cases in which closure seems to influence perceived extent. Inspired by a famous “bar bet,” Rivera-Aparicio and Firestone (2018) demonstrated that the circumference of a closed circle is perceived by as much as 35% shorter than a similar but not-fully-closed shape (see Fig. 2E). This *pint glass illusion* is particularly compelling because it is so simple that it leaves little room for counter-explanation. Thus, it is clear that closure must play some role in perceived extent, though exactly how and when and why this happens remains a mystery.

### Distortions of multidimensional space

**Fig. 3:**
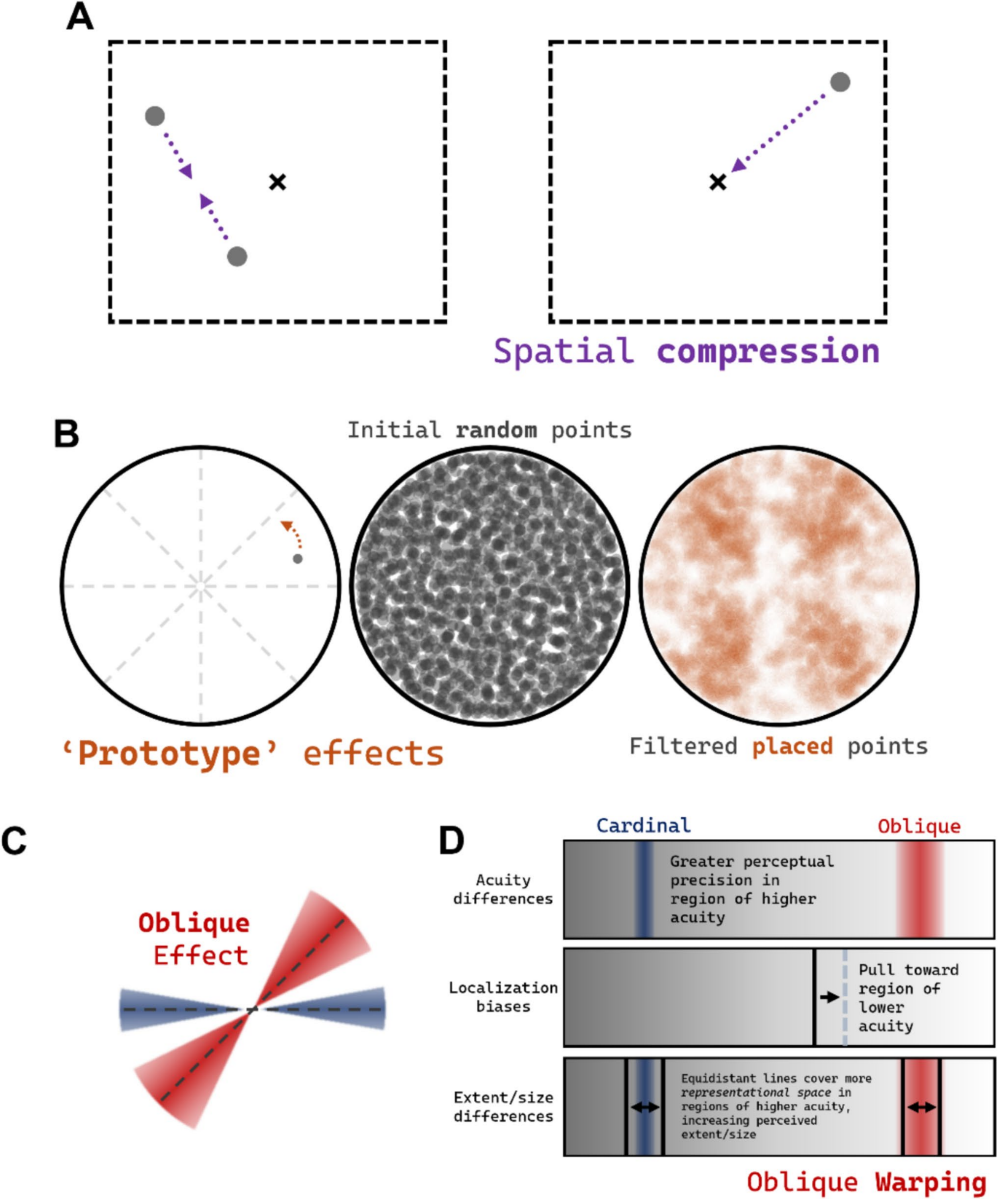
Illusions of spatial location. **A** Two examples of spatial compression, wherein locations are either compressed toward each other or toward a center point. **B** An example of prototype effects wherein objects are mislocalized toward the centers of their respective quadrants (using data from Yousif et al., 2020). **C** The classic oblique effect whereby oriented bars in the oblique region are harder to perceive. **D** A visual explanation of oblique warping, which describes various other oblique distortions. (Color figure online)

Most of the effects discussed so far concern distortions of space in a single dimension (e.g., along a single axis). In reality, of course, much of our interaction with the world occurs in (at least) two dimensions. How is multidimensional space distorted? Perhaps the simplest two-dimensional spatial distortion is *spatial compression*, demonstrated by Sheth and Shimojo (2001). They showed that objects in memory are robustly compressed toward the center of gaze and/or toward other salient landmarks (see Fig. 3A). This compression is robust in memory, though it is unclear whether these effects are also perceptual in nature—mostly because this is impossible to demonstrate empirically. For example, you might think of the empty-space condition used by Vickery and Chun (2010) as a test of perceptual spatial compression, but what experimental control could be used to claim that compression occurs? Perhaps the distance between two dots could be compared against the distance between the two endpoints of a line (see Experiment 1b of Yousif & Scholl, 2019). But even then, it would not be clear whether the space between the two dots is being compressed, or the space of the continuous line is being expanded. Perhaps both are true. In the absence of any definitive evidence, we think it is reasonable to assume that spatial compression likely occurs in perception as it does in memory, given the fact that we already know continuous entities are perceived as longer than discrete entities.

Two-dimensional space can be distorted in much more complex ways, however. Consider, for instance, *prototype effects—*perhaps the most well-established spatial distortion of all—wherein locations are misremembered with respect to the regions in which they are situated (Huttenlocher et al., 1991). Imagine any dot inside of a circle, as in Fig. 3B. Suppose the dot disappears and you must replace it within the shape. Chances are that you will make the same mistake that everyone does: You will misplace the dot farther toward the center of its original quadrant. These sorts of effects have been documented in all sorts of environments (Holden et al., 2010), including in three-dimensional space (Holden et al., 2013). Huttenlocher and colleagues explained these effects by appealing to work on concepts and categorization showing that memories are distorted toward their prototypical form (e.g., Bartlett, 1932; Posner & Keele, 1968). That is, any memory you have of any one car will be biased to resemble an average, *prototypical* car. They argued that spatial memory works the same way: Objects are segmented into natural categories (e.g., quadrants) and, as a result, memories for locations within those objects are biased toward the prototype (i.e., center) of that category. If this story is accurate, it would suggest that memories for all locations are distorted with respect to the frames that they occupy.

Prototype effects are not just effects of memory, however. Even in speeded perceptual tasks, people make the same sorts of systematic errors: They are slower and less accurate when discriminating locations toward the centers of quadrants (Yousif et al., 2020). Yet, unlike Huttenlocher and colleagues, Yousif and colleagues (2020) do not see errors as biases toward the center of the quadrants—not exactly. They point out that these biases only exist for *angular* changes, not *distance* changes. That is: The perception of space is distorted as a function of an object’s proximity to the oblique axes, but only in the angular dimension. If an object shifts toward or away from the center of a circle, even if it is right along the oblique axis, perceptual acuity is not impaired.

The dissociation between errors in the angular and distance dimensions of space is consequential; it may suggest that locations are naturally represented in polar coordinates. Further analyses of these *prototypical* biases revealed further support for this possibility (see Yousif & Keil, 2021). In fact, it turns out that these biases are remarkably general; they span not only memory and perception but also multiple kinds of spatial information. For example, one of the most well-known psychophysical effects is the *oblique effect*, which, on its surface, is a lot like *prototype effects*. Whereas prototype effects (and the additional angular biases discovered by Yousif and colleagues) involve mislocations toward and lower acuity near the oblique axes, the oblique effect describes reduced acuity for oriented lines in those same regions of space (see Appelle, 1972; Bonds, 1982; Essock, 1980; see Fig. 3C). One often-unappreciated fact is that these oblique distortions are quite general in nature—extending far beyond just orientation and even beyond location. It turns out that the *sizes* of objects, and the distances between them, are also distorted, such that things appear less extended when they span the oblique regions as opposed to the cardinal regions (Yousif & McDougle, 2024).

Angular biases are not limited to the visual modality; they reflect a more general distortion of space. Yousif, Forrence et al., (2024a) showed that similar biases manifest in a purely motor task, without any visual input whatsoever. In fact, the magnitudes of the visual and non-visual biases are correlated: A person who makes larger mislocalizations toward the oblique regions of space in a visual task will also make larger mislocalizations in a nonvisual task, independent of the overall amount of error that they make (Yousif, Forrence et al., 2024a). Relatedly, deficits in visual acuity for locations in the oblique regions of space are correlated with deficits of orientation perception (Yousif & McDougle, 2024), leading to the suggestion that all of the aforementioned effects may be linked (see Fig. 3D for a visual explanation). That is, space itself may be warped in the oblique regions of space, so that there is reduced acuity in those regions. As a consequence of this reduced acuity, memories for locations may be pulled toward these regions, resulting in well-known biases like *prototype effects*. Similarly, spatial extent is reduced in the oblique regions because those regions span less *representational space* (on account of their decreased sensitivity). Yousif and McDougle (2024) call this general distortion in the oblique regions of space *oblique warping*.

Crucially, *oblique warping* offers a way to understand classic effects of mislocalization (e.g., Huttenlocher et al., 1991) without appeal to processes of categorization. Prototype effects may arise not because of a categorical division between the different quadrants of space, but because of continuous variation in visual acuity throughout space. Or both of these things may be true at once: There may be continuous variation in visual acuity and *also* top-down, categorical biases that influence our sense of space. For now, it remains unclear.

Note that others have claimed precisely the opposite—that objects in spatial memory are mislocalized toward regions of *higher* acuity (Langlois et al., 2021). Yet the stimuli used in the two cases are remarkably different: Whereas Yousif and McDougle (2024) theorize about spatial representation within contained environments (like within a circle, per the original work of Huttenlocher et al., 1991), Langlois and colleagues (2021) study spatial memory for dots superimposed on everyday images—like a picture of a lighthouse, for instance. When they examined various canonical shapes, they did find biases toward the edges of shapes (regions of high acuity) but they *also* found biases toward the oblique regions of those very same shapes (regions of low acuity). It is not clear how both of these biases co-exist. One possibility is that the former bias of spatial localization relies on explicit visual landmarks, whereas the latter may rely more on spatial processes like those involved in spatial navigation. Future work may seek to explain both sorts of biases in tandem.

Combine all of this together, and it is easy to imagine that our mental representation of the spatial world is being constantly pushed and pulled in various directions, as if dozens of gravitational forces are in effect at once. Yet, as we have discussed, these distortions are not arbitrary. The spatial world is systematically warped with respect to the objects that fill it.

## Distortions of time in and around events

How long did your morning meeting last? Did you eat lunch before or after your class? When we are faced with questions like when something occurred or how long it lasted, we often find that our sense of time powerfully diverges from reality. Why might this be the case? In this section, we argue that in the same way that our representations of space are distorted by the presence of objects, our experience of time—on both short and long timescales—is distorted by proximity to events, leading to powerful and robust illusions of time in both perception and in memory.

### Distortions of time caused by the bounds of an event: Analogs to object-based effects

**Fig. 4:**
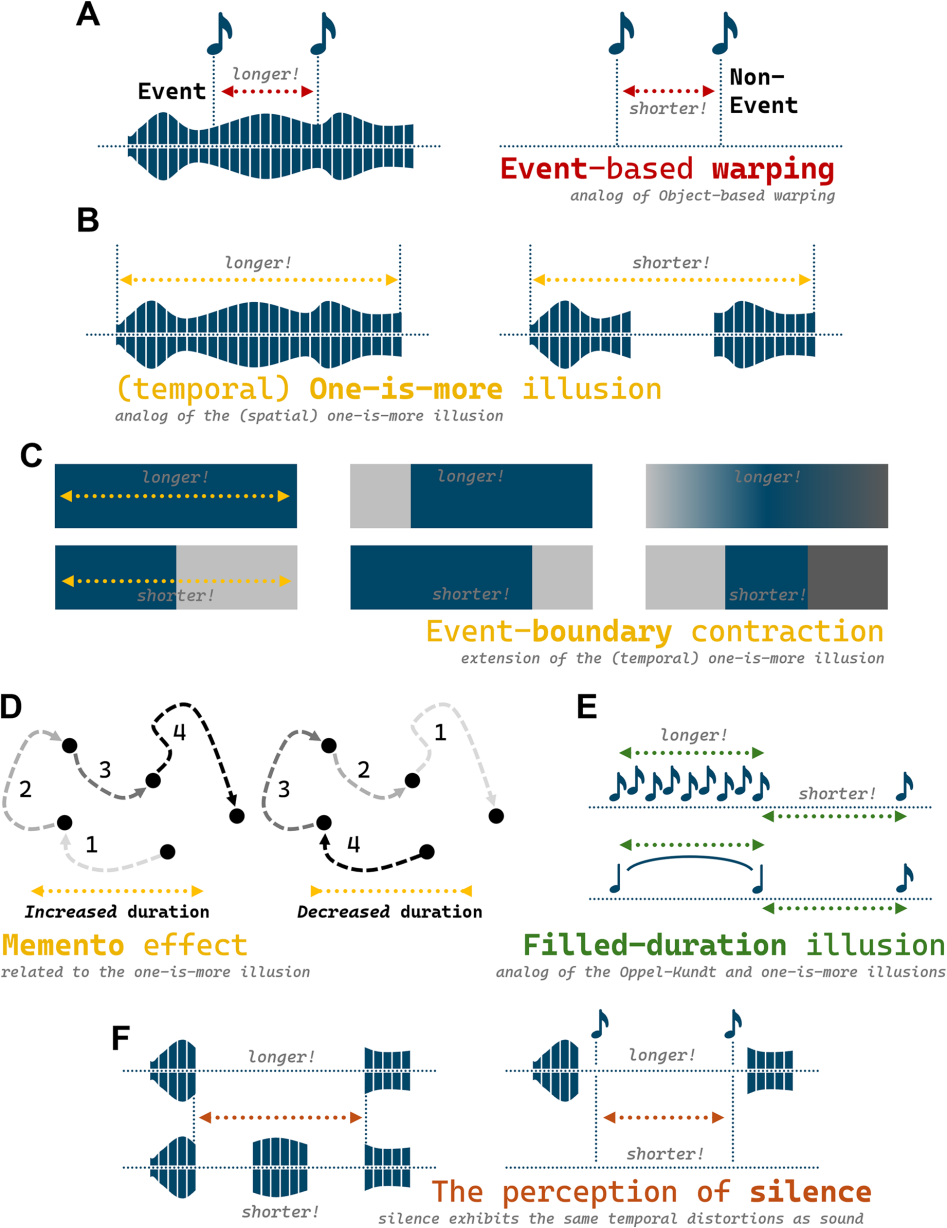
Temporal analogs of illusions of spatial extent. **A** Event-based warping, wherein probes within a continuous event appear further apart in time. **B** The (temporal) one-is-more illusion, wherein continuous periods of time are perceived as longer than discontinuous ones. **C** Variations and extensions of the one-is-more illusion, or event-boundary contraction. **D** The “Memento” effect, wherein events played in a reverse order are perceived as shorter in duration. **E** Two variations of the filled-duration illusion wherein the filled durations are perceived as longer. **F** Illustrations of how these illusions apply to silence as well as sound. In all cases, the relevant extents are highlighted with dotted lines. (Color figure online)

Virtually all of the object-based, spatial distortions discussed above have an event-based, temporal analog. For instance, Goh et al. (2025) demonstrated that just as two dots within an object are perceived as farther apart in space, two tones within a continuous auditory event are perceived as further apart in time—*event-based warping*. For them, an auditory event was something like the background noise that one might experience while sitting in a coffee shop. Simply: Two tones that played during a segment of background noise were perceived as further apart than two tones that played independent of such a segment (see Fig. 4A). They conducted several additional experiments to ensure that the effects were about eventhood *per se*. For instance, they demonstrated that the effects could not be explained by the onset or offset of background noise alone.

As with objects, it is not just that time is distorted by the presence of events; events *themselves* also seem to be distorted: The *one-is-more illusion* also has a temporal analog. Yousif and Scholl (2019) showed that continuous periods of noise were perceived as substantially longer than discrete moments of noise interrupted by a period of silence—on average, by about 30% (see Fig. 4B). Just like its spatial counterpart, the auditory one-is-more illusion persists even when an “auditory occluder” is used to create a gap without disrupting the underlying continuous tone. Sherman and colleagues (2023), exploring the role of memory in the perception of time, independently discovered the same sort of illusion. In addition, they showed that the illusion is impacted by *when* a salient event boundary occurs and that abrupt changes may have a greater impact on perceived time than gradual ones (see Fig. 4C). And according to Singhal and Srinivasan (2022), these sorts of temporal distortions can result not just from *externally* defined events, but internal perceptual switches—as occurs, for instance, when one swaps from one interpretation of an ambiguous Necker cube to another.

These findings also resemble an earlier finding from Liverence and Scholl (2012), who demonstrated that continuous paths are perceived as longer in duration than discrete ones. To control various factors like predictability and attention, the discrete paths consisted of four segments of the continuous path played in reverse order (such that the fourth segment played first, then the third, etc.; see Fig. 4D). (Referencing the 2000 Christopher Nolan thriller *Memento*, they called these “Memento” trials.) In this way, they were able to isolate the difference in perceived duration that arose from the segmentation of the events *per se*. This *Memento effect* is also unique in that, unlike most of the other illusions and distortions discussed in this section, it involves visual segmentation—highlighting a special case of how *spatial* objects (in this case, paths) can influence *temporal* processing. However, there has been some debate about whether this effect reflects event processing per se. Meyerhoff and colleagues (2015) later argued that the findings are better explained by spatiotemporal unpredictability in the discrete-event condition. (Of course, one might then wonder how different “spatiotemporal unpredictability” and “events” really are. Surely spatiotemporal variability is one of the key cues to eventhood in the first place; see Yates et al., 2023)

The Oppel–Kundt also has a temporal analog in the *filled duration illusion*. Just as filling the space between two lines with more lines creates an impression of increased spatial extent, filling the gap between two tones with more tones creates an impression of temporal extent (Thomas & Brown, 1974; Wearden et al., 2007; see Fig. 4E). Variants of the filled-duration illusion better resemble the one-is-more illusion, however: Periods filled by a single continuous sound are also said to be perceived as longer in duration than periods between two discrete sounds (see Fig. 4E).

Another class of temporal distortions involves *oddball effects* (Tse et al., 2004), whereby one’s sense of time is expanded for unexpected events. That is, if you see a circle in the middle of a sequence of squares, you will perceive that circle as having lasted longer. While this effect is not directly analogous to any of the object-based spatial effects discussed earlier, it is still deeply related to those effects (and similar event-based temporal effects) via *attention*. As Tse and colleagues (2004) succinctly put it: “The perception of duration is rooted in the perceptual processing of events” (p. 1171). Their idea is that time ought to slow down for the unexpected, so that such events have more (subjective) time to be processed, as if more temporal attention is allocated to it. Perhaps this is not unlike the way that individual objects appear larger in space when spatial attention is allocated to them (e.g., Yousif & Scholl, 2019).

Since its discovery, many variations of the oddball effect have been documented (for review, see Eagleman, 2008). Particularly notable (though perhaps not particularly surprising) is the fact that oddball stimuli *anywhere* in the visual environment seem to expand the sense of time for everything else in the display, suggesting that the phenomenon is more about the *event* caused by the surprise, rather than about the surprising object itself (New & Scholl, 2009). That is, events, independent of the particular entities comprising them, are the fundamental unit of our sense of time.

One reason to believe that these effects genuinely reflect *eventhood* (as opposed to just the presence of sensory information) is that many of them apply to *silence* as well as sound. Goh et al. (2023) demonstrated that event-based warping, the one-is-more illusion, and oddball effects on time perception all work the same way when periods of silence are substituted for periods of sound. That is: Extended segments of silence are temporally extended, and tones within those periods of silence are perceived as further apart in time (see Fig. 4F). These findings suggest that what the mind represents is not just auditory stimulation, but meaningful *units of time—*whether those units are characterized by the presence of something or its absence.

The fact that these illusions are about eventhood rather than sound per se gives us a new lens through which to see some of these temporal illusions. For example, you might notice that the *filled-duration illusion* and *event-based warping* are essentially the same phenomenon—except that, in the latter case, we now know that the effect also works for periods of silence rather than sound. Thus, it seems that the *filled-duration illusion* is poorly named, since the illusion itself is not caused by filled durations at all; “empty” durations can also be expanded, so long as those empty periods are perceived as meaningful units of time.

### Distortions of time by event hierarchy

Work on object perception mostly distinguishes between complete objects (like the single rectangle of the one-is-more illusion; see Fig. 1D) and separate objects (like the two discrete rectangles in the same illusion; see Fig. 1D). But the Oppel–Kundt illusion shows us that the perception of space is also influenced by the number of intervening entities (see Fig. 2B). Or, alternatively, the intervening vertical bars cause the outer bars to be perceived as part of one coherent, striped object, such that the Oppel–Kundt illusion essentially becomes a variation of the one-is-more-illusion. Formally, it is not clear whether or when a group of items ought to be recognized as one coherent object. In the world of temporal memory, though, it is understood that events are hierarchical and can be composed of other events (see Yates et al., 2023). Indeed, many paradigms in event cognition involve establishing boundaries between periods that contain many discrete image presentations (see, e.g., Clewett et al., 2020; DuBrow & Davachi, 2013, 2016; Heusser et al., 2018). Yet in such cases, it is perfectly normal to refer to those periods as “events.” In fact, what tends to be emphasized in the event perception literature is not the events themselves, but the boundaries *between* them.

In practice, this appreciation of the hierarchical nature of events allows for the study of events to span multiple timescales. Work has investigated temporal memory distortions on the scale of seconds (e.g., most of the work reviewed above), minutes (e.g., DuBrow & Davachi, 2013), days (e.g., Sherman & Yousif, 2025), and even years (e.g., Yousif, Lee et al., 2024b). One idea pervades this entire literature—that, somehow or another, temporal memory is distorted at event boundaries.

**Fig. 5:**
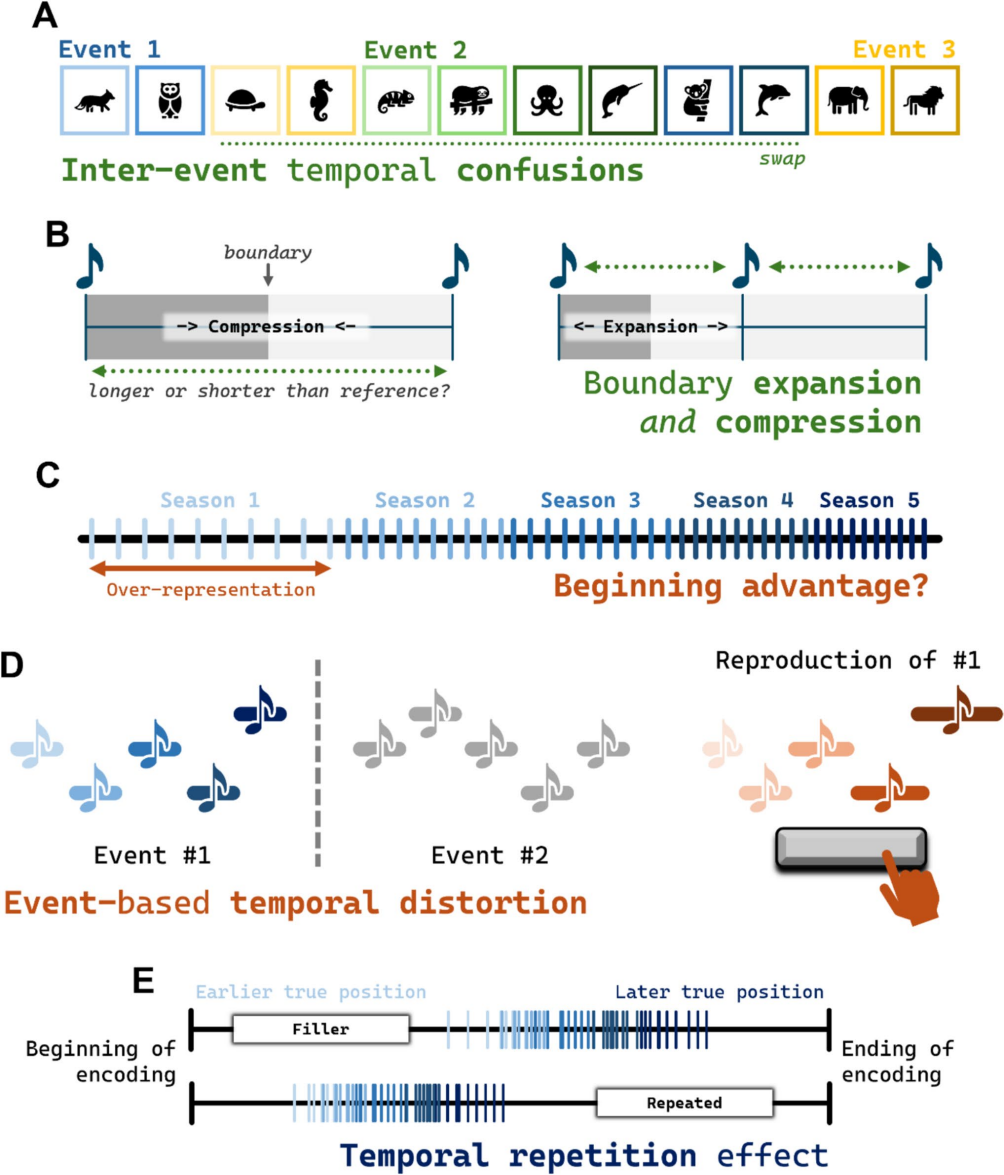
Other event-related temporal distortions. **A** An illustration of temporal confusions caused by event boundaries. **B** Two contradictory temporal distortions resulting from different time perception manipulations (per Bangert et al., 2020). **C** An illustration of a “beginning advantage” whereby beginnings are overly represented on mental timelines (per Yousif, Lee et al., 2024b). **D** An example of an event-based temporal distortion, whereby beginnings are compressed and endings are expanded (per Wen et al., 2025). **E** The temporal repetition effect wherein repeated items are perceived as initial occurring longer ago (per Sherman & Yousif, 2025). In all cases, the relevant extents are highlighted with dotted lines. (Color figure online)

For example, DuBrow and Davachi (2013) showed that, on the scale of seconds or minutes, boundaries disrupt temporal order judgments such that memory for the order of items *within* an event is better preserved than memory for the order of items *across* events (see also Clewett et al., 2020; DuBrow & Davachi, 2016; Heusser et al., 2018; see Fig. 5A)—a classic event boundary effect. However, Yousif, Lee, and colleagues (2024b) found the exact opposite when examining event representation “at the scale of ordinary experience.” In their study, they probed memory for events for popular television shows that were experienced not on the scale of seconds or minutes, but months and years. For the longest-running show tested, participants would have first viewed that material over a *decade* prior to testing. In this task, when placing events on a timeline, participants were more likely to confuse events within a season than across seasons (otherwise equating for temporal distance). The authors speculate that this is not because of a difference in timescale (compared with the classic work of DuBrow and Davachi) but a difference in how the relevant sequences were structured. In the work of DuBrow & Davachi (2013), the sequences were arbitrary; there was no coherent narrative order to them. That is quite unlike the rich, structured narratives contained in television shows. In the latter case, it makes sense that participants would be less likely to make across-season confusions. Wen and Egner (2022) showed exactly this—increased *within* event confusions for sequences with meaningful temporal structures, even at the same temporal scale as the original findings from DuBrow and Davachi. For this reason, we do not see these opposing results as conflicting with one another. Instead, they demonstrate that the effects of event boundaries on temporal memory are context- (and perhaps content-) sensitive. Further, regardless of the directionality of the effect (and regardless of the reason for the differences), it remains clear that event boundaries do affect our ability to reconstruct the temporal order of our experiences. In this way, it is clear that event boundaries play a role in temporally organizing our memories.

Relatedly, perceived and remembered duration can also be influenced by event boundaries. For example, one way to interpret the results of the temporal one-is-more-illusion (along with related findings; e.g., Sherman et al., 2023; Liverence & Scholl, 2012) is that the presence of an event boundary caused a compression of perceived time. Intriguingly, these findings stand in contrast to a wealth of findings that event boundaries can lead to an expansion in temporal *memory –* that is, intervals containing an event boundary are judged as longer than comparable intervals without a boundary (Clewett et al., 2020; DuBrow et al., 2024; Ezzyat & Davachi, 2014). Bangert and colleagues (2020) showed people naturalistic movies of everyday events (see Fig. 5B) and found that the directionality of the relationship between boundaries and time can be impacted by how exactly duration estimates were probed. When participants were asked to compare durations across boundaries to a fixed duration held in long-term memory, events spanning a boundary were perceived as shorter in duration. Yet when participants were asked to compare whether a middle tone was (temporally) closer to a first tone or a third tone, an intervening boundary caused the tones to be perceived as further apart from one another. Bangert and colleagues (2020) argue that the most parsimonious explanation of the discrepancy is a difference in how attention is deployed in the two cases. We won’t adjudicate this (nor whether such a distinction could explain the opposing results in shorter-term perception vs. long-term memory) here. What matters for our purposes is that, even though there can be opposing effects of boundaries on representations of time, there are several independent lines of work which clearly implicate event boundaries in temporal processing.

Finally, some recent work has suggested that it’s not just *durations* of events (or items within an event) that can be distorted, but that the relative timing (or rhythm) of items within an event can be altered by temporal structure. Specifically, Ongchoco et al. (2023) designed a task in which participants reproduced musical sequences. They found that participants reliably “sped up” prior to an event boundary (playing the notes prior to a boundary earlier than they should) and “slowed down” following an event boundary (playing the notes following a boundary later than they should). These data suggest events not only play a role in shaping overall judgments of duration or order, but also may alter the way we represent the precise temporal relationships among items within vs. across events.

Events are not just about boundaries, however. Responding to this observation, Yates and colleagues (2023) point out that the focus should instead be on the substance of the events themselves. “A room is more than just four walls,” they write, “Characterizing a room has more to do with what exists between the walls than it does with the walls themselves” (p. 2077). They argue that events are the same way, and that, accordingly, more emphasis should be placed on the *internal* structure of events—their beginnings, middles, and endings.

In this spirit, Yousif, Lee, and colleagues (2024b)—in the same study analyzing temporal memory errors in popular television shows—explicitly examined how beginnings and endings of narratives are relatively expanded or compressed. In the case of *Game of Thrones*, for instance, they showed that events that occurred in Season 1 were regularly placed much further along the timeline, as if the beginning of the show was overrepresented on people’s mental timelines (see Fig. 5C). This was true even when the boundaries between seasons were explicitly labeled, suggesting that this finding was not just a matter of misrepresenting the scale of the timeline itself. In fact, this is an effect that readers may be able to appreciate for themselves: If you ever rewatch a show after some time, you may notice that prominent events early in the narrative occur *sooner* than you expect. This, we think, is naturally explained by the fact that beginnings are overrepresented on the mental timeline. Of note, these findings are consistent with other work documenting other sorts of “beginning advantage[s]” in the representation of events (Teigen et al., 2017).

That said, the relationship between the internal structure of events and perceived duration is not conclusively established. Studying time perception on shorter timescales, Wen and colleagues (2026) showed that beginnings were remembered as being relatively shorter and endings were remembered as being relatively longer. Critically, they showed that these effects are explained by event structure per se and not merely primacy and recency. In one experiment, participants listened to *two* sequences of tones, one after the other; the authors showed that the effects of beginnings/endings applied even to the first sequence (despite the fact that its ending was relatively further away in time; see Fig. 5D). Future work may wish to unpack how these findings relate to findings on longer timescales (e.g., Yousif, Lee et al., 2024b).

So far, we have discussed ways that impressions of time may be shaped by *event boundaries* and *the structure of the event itself* (i.e., its beginning, middle, or ending). One final factor that may distort the sense of time is *repetition* of the event itself. Classic models of memory posit that, for example, repeated events will have greater memory strength, and thus that such events will be remembered as relatively recent (Hintzman, 2005; see also Flexser & Bowser, 1974; Hintzman, 1988, 2004, 2010; Zou & Kuhl, 2024). However, Sherman and Yousif (2025) show that this is not always true. When people are asked to recall not when an event most *recently* occurred, but when it *first* occurred, they tend to recall repeated events as occurring *earlier* in time (see Fig. 5E). And this is true not only on the scale of minutes, but *days*: Participants exhibited the same bias even when they were tested over the course of one full week. This *temporal repetition effect* is not readily explained by existing memory models. This finding is not straightforwardly explained by explicit strategy on behalf of the participants, either: When asked whether they used the number of remembered repetitions as a heuristic to place items on the timeline (i.e., whether they placed something further back because they reasoned they must have seen it longer ago, on account of remembering it multiple times), participants reported a wide range of responses. Critically, though, these explicit reports of heuristic strategies were uncorrelated with the effect itself. Sherman and Yousif note that this does not fully rule out the possibility of heuristic strategies, but it does suggest that the temporal repetition effect is not *obviously* explained by such strategies. There is room for future work to investigate this matter further.

## Object and events, space, and time—Inextricably intertwined?

There is no denying that there is a deep relationship between the representation of objects and events, space and time. However, despite all the similarities, there are some substantive differences, as well. In this section, we will briefly comment on the similarities before discussing some of those differences and their implications.

Dozens of papers explicitly compare the representation of objects and the representation of events (see, e.g., De Freitas et al., 2014; Lee et al., 2024; Papafragou & Ji, 2023; Wellwood et al., 2018; Yates et al., 2023; Yousif & Scholl, 2019). Both events and objects are discretizations of otherwise continuous input. Both objects and events seem to influence—or be influenced by—attention in similar ways (see, e.g., De Freitas et al., 2014; Goh et al., 2025; Yousif & Scholl, 2019). And as we have discussed throughout this paper, both events and objects seem to expand time and space in similar, systematic ways.

Even so, there are a number of subtle differences between the two that are worth mentioning. For example, readers may note that while our usage of the term “object” was straightforward (but see Baker et al., 2024), our usage of the term “event” was not. That is, the word *event* (here and elsewhere) is sometimes used to refer to a specific *moment* and other times used to refer to extended *periods* (for more on this distinction, see Yates et al., 2023). The effects discussed here encompass both moments and periods. Yet it is not clear what the spatial equivalent would be of these terms. More than just wordplay, these distinctions affect how these entities are studied. For example, it is natural to think about events embedded within events—like scenes within episodes within seasons of a television show (Yousif , Lee et al., 2024b). It is understood, in other words, that events are *hierarchical*. While objects obviously can be embedded within other objects, we are not aware of any case in which a study relied on hierarchical embeddings of objects in a systematic way.

On the contrary, it is natural in the domain of space to distinguish between *objects* and *substances* (see, e.g., Soja et al., 1991; VanMarle & Scholl, 2003)—but what is the temporal equivalent of a substance? Background noise? Perhaps it depends on context. In Goh and colleagues’ (2025) study of event-based warping, ambient noise was treated as a canonical auditory event. Relative to absolute silence, perhaps this qualifies as an event, but in other contexts, this assumption would surely be rejected. This difference matters only insofar as we want to make comparison to the spatial equivalents: If we really want to understand the similarities between object and event representation, we may want to first ensure that we are comparing comparable entities.

Then there is the matter of *absence*. In the spatial domain, we can think of holes as a kind of absence (see, e.g., Nelson & Palmer, 2001). In the temporal domain, we can think of silence as a kind of absence (Goh et al., 2023). But absences can occur not only between objects and events; absences can also exist because nothing is present at all. How are those absences represented—and are there any commonalities between objects and events in that respect? Short of staring at an empty wall, there is really no way of thinking about what it means to have an object-less impression of the spatial world. The temporal world is a bit different, though: There are many moments in between conversations and thoughts that are more than just pauses, when the mind seems to be in no particular moment and attending to no particular thing. For now, it remains an open question whether these sorts of absences are comparable to one another and whether they have similar cognitive consequences.

Another notable distinction in our discussion of objects and space versus events and time is that whereas the majority of spatial distortions have been studied within the domain of perception, much of our understanding of how events influence representations of time come from studies of memory (e.g., DuBrow & Davachi, 2013; Sherman & Yousif, 2025; Yousif, Lee et al., 2024b). In part this is a necessity: Because events dynamically unfold over time, judgments of time are almost always retrospective, and thus might involve memory to some extent. In contrast, because distortions of space can be observed and measured during perception itself, it is more natural to study such distortions then. However, work in the time/event domain does suggest that there may be interesting distinctions between perception and memory: For example, event boundaries can exert opposing effects on short-term, in-the-moment judgments of time (e.g., Sherman et al., 2023; Yousif & Scholl, 2019) versus longer-term memory (e.g., Ezzyat & Davachi, 2014), raising interesting questions about how event-based distortions of time change *over time*. There is the opportunity to explore similar questions in the spatial domain: Whereas there is some evidence suggesting that some spatial distortions are similar across perception (Yousif et al., 2020) and memory (Huttenlocher et al., 1991), there is also some evidence that spatial biases can change over time. For example, in a virtual navigation task, Graves and colleagues (2020), found that although participants initially navigated toward specific learned locations in an arena, they subsequently navigated based on the *distribution* (i.e., they were more likely to traverse to the average location, rather than to specific locations; see also Graves et al., 2022). Thus, future work might explore whether and how the object-induced spatial distortions discussed here may change with memory. For example, do the distortions become increasingly strong over the course of memory consolidation?

One final subtle difference between objects and events is that events are intrinsically more dynamic: As time unfolds and we acquire new information, our understanding of the past is transformed. New chapters of our lives, or of human history (see Teigen et al., 2017), may be recognized only in retrospect. This is true on short timescales as well as long ones. For instance, events after an ambiguous collision can cause a *prior* event to appear more causal in retrospect (Choi & Scholl, 2006). Whether or not the dynamic reshaping of events in memory is of any consequence remains to be seen, however; this is another matter for future work.

Despite these differences, it is clear that the relationship between the representation of objects and events is an important one. After all, these concepts bear on the nature of our experience itself—how it is that the complex, noisy world around us is carved into discrete perceptions of space and time. That objects and events exert similar influence on our perceptual experience—in ways that are substantially and readily perceptible in simple demonstrations!—is a testament to their enduring similarity.

## Conclusion

Everything that we do and everything that we experience is inexorably influenced by the gravity of objects and events. Here, we have reviewed dozens of distortions of space and time that result directly from it, many of which appear to be clear analogs of one another. Similarities in the representation of objects and events point to an underlying cognitive architecture that represents the world in discrete units, perhaps with some common systems that extend across modalities and domains. Even so, there remain subtle differences between objects and events—and the distortions that arise from them—that foreshadow many inspiring questions yet unanswered.

## Data Availability

Not applicable.
